# Weak inter­actions in the crystal structures of two indole derivatives

**DOI:** 10.1107/S2056989016008616

**Published:** 2016-06-17

**Authors:** Jamie R. Kerr, Laurent Trembleau, John M. D. Storey, James L. Wardell, William T. A. Harrison

**Affiliations:** a Department of Chemistry, University of Aberdeen, Meston Walk, Aberdeen AB24 3UE, Scotland; b Fundação Oswaldo Cruz, Instituto de Tecnologia em Fármacos-Far Manguinhos, 21041-250 Rio de Janeiro, RJ, Brazil

**Keywords:** crystal structure, indole, N—H⋯O hydrogen bonds, inversion dimers, weak inter­actions

## Abstract

We describe the syntheses and crystal structures of two indole derivatives, namely a second monoclinic polymorph of ethyl 5-chloro-1*H*-indole-2-carboxyl­ate C_11_H_10_ClNO_2_, (I), and ethyl 5-chloro-3-iodo-1*H*-indole-2-carboxyl­ate, C_11_H_9_ClINO_2_, (II). In their crystal structures, both compounds form inversion dimers linked by pairs of N—H⋯O hydrogen bonds, which generate *R*_2_^2^(10) loops. The dimers are linked into double chains in (I) and sheets in (II) by a variety of weak inter­actions, including π–π stacking, C—I⋯π, C—Cl—π inter­actions and I⋯Cl halogen bonds.

## Abstract 2

The weak inter­molecular inter­actions in two indole derivatives are described.

## Chemical context   

As part of our ongoing synthetic, biological (Kerr, 2013 ▸) and structural studies (Kerr *et al.*, 2016 ▸) of variously substituted indole derivatives, we now report the syntheses and crystal structures of ethyl 5-chloro-1*H*-indole-2-carboxyl­ate (I) and ethyl 5-chloro-3-iodo-1*H*-indole-2-carboxyl­ate (II), which differ in the substituent (H or I) at the 3-position of the ring system. Compound (I) is a second monoclinic polymorph of the recently described 5-chloro-1*H*-indole-2-carboxyl­ate (Wu *et al.*, 2013 ▸).
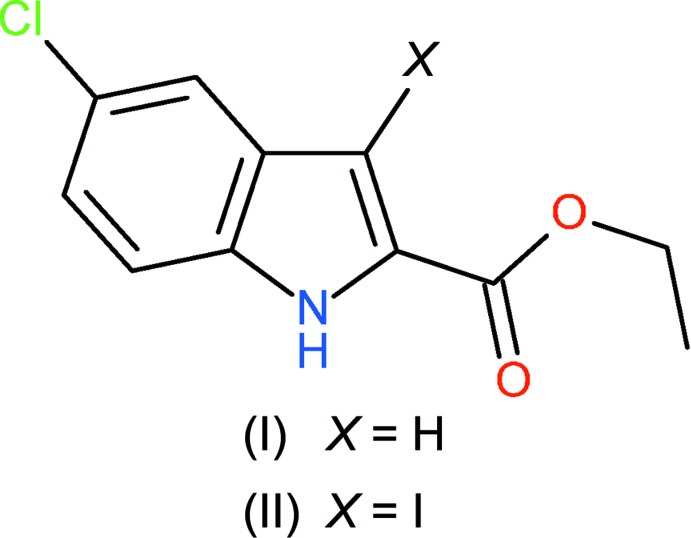


## Structural commentary   

**Figure 1:**
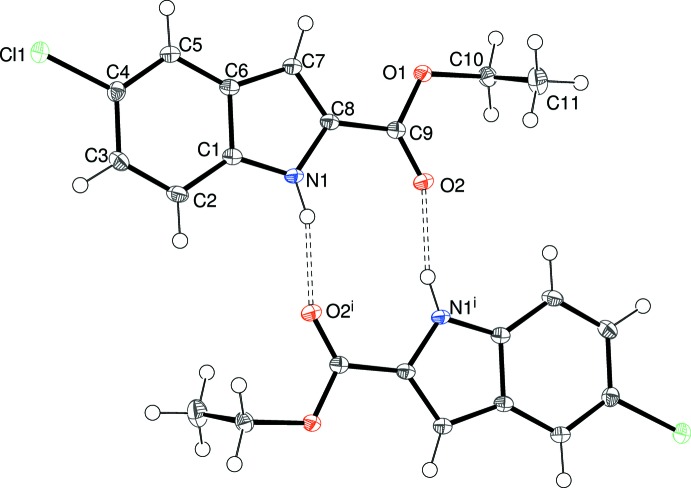
The mol­ecular structure of (I) showing 50% displacement ellipsoids. Also shown with double-dashed lines are the pair of inter­molecular N—H⋯O hydrogen bonds to a nearby mol­ecule related by inversion symmetry, which generate an 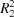
(10) loop. Symmetry code: (i) 1 − *x*, 2 − *y*, 1 − *z*.

Compound (I) crystallizes in space group *P*2_1_/*n* with one mol­ecule in the asymmetric unit (Fig. 1 ▸). The dihedral angle between the mean plane of the N1/C1–C8 indole ring system (r.m.s. deviation = 0.010 Å) and the C9/O1/O2 grouping is 2.4 (2)°. The chlorine atom deviates from the indole plane by 0.0625 (14) Å. The C8—C9—O1—C10 torsion angle of −178.86 (11)° indicates an *anti* conformation about the C9—O1 bond, whereas the C9—O1—C10—C11 torsion angle is −81.73 (14)° and C11 projects from the mean plane of the other non-hydrogen atoms by 1.298 (2) Å.

In the structure reported by Wu *et al.* (2013 ▸), (CCDC refcode VIHMUW), the same mol­ecule also crystallizes in space group *P*2_1_/*n* [*a* = 10.570 (3), *b* = 5.6165 (15), *c* = 18.091 (5) Å, β = 105.681 (4)°, *V* = 1034.0 (5) Å^3^, *Z* = 4]: the only significant conformational difference compared to (I) is (using our atom-labelling scheme) the C9—O1—C10—C11 torsion angle of 173.19 (12)°, which indicates that the mol­ecule in the Wu *et al.* polymorph is almost planar (r.m.s. deviation = 0.031 Å for 15 non-hydrogen atoms). The densities of (I) [ρ = 1.438 g cm^−1^] and the Wu polymorph [ρ = 1.437 g cm^−1^] are essentially identical.

**Figure 2:**
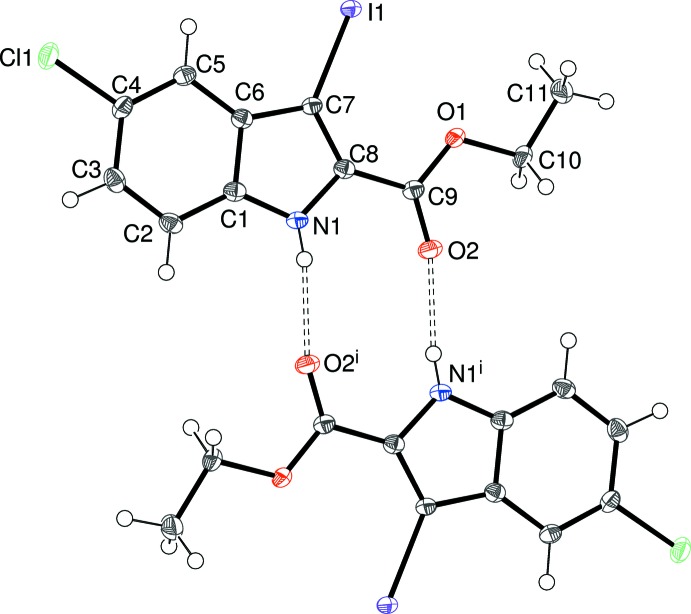
The mol­ecular structure of (II) showing 50% displacement ellipsoids. Also shown with double-dashed lines are the pair of inter­molecular N—H⋯O hydrogen bonds to a nearby mol­ecule related by inversion symmetry, which generate an 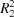
(10) loop. Symmetry code: (i) −*x*, 2 − *y*, 1 − *z*.

There is one mol­ecule in the asymmetric unit of (II), which crystallizes in space group *P* 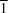, as shown in Fig. 2 ▸. The C9/O1/O2 grouping is almost coplanar with the mean-plane of the indole ring system (r.m.s. deviation = 0.009 Å), as indicated by the dihedral angle of 3.95 (7)° between C1–C8/N1 and C9/O1/O2. Atoms Cl1 and I1 deviate from the indole plane by −0.106 (2) and 0.081 (2) Å, respectively. The conformation of the C8—C9—O1—C10 bond in (II) [torsion angle = −177.42 (16)°] is almost the same as the equivalent grouping in (I), but the C9—O1—C10—C11 torsion angle of −178.33 (17)° is quite different, and indeed, the complete mol­ecule of (II) is almost planar (r.m.s. deviation = 0.033 Å for 16 non-hydrogen atoms).

## Supra­molecular features   

**Figure 3:**
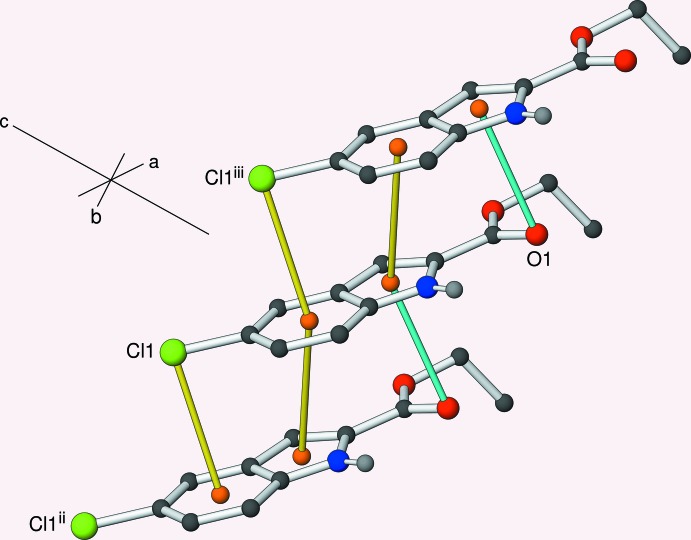
Partial packing diagram for (I), showing the formation of [010] chains linked by π–π and C—Cl⋯π inter­actions (yellow lines). The long C=O⋯π contact is indicated by a cyan line. All hydrogen atoms except H1 are omitted for clarity. Symmetry codes (ii) *x*, *y* + 1, *z*; (iii) *x*, *y* – 1, *z*.

In the crystal of (I), inversion dimers linked by pairs of N—H⋯O^i^ [symmetry code: (i) 1 − *x*, 2 − *y*, 1 − *z*] hydrogen bonds (Table 1 ▸, Fig. 1 ▸) generate 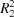
(10) loops. The first weak inter­action to consider is aromatic π–π stacking between the C1–C6 (π_6_) ring and the C1/C6/C7/C8/N1 (π_5_) five-membered ring displaced by translation in the *b*-axis direction (Fig. 3 ▸). The π_6_–π_5_^ii^ [symmetry code: (ii) *x*, 1 + *y*, *z*] centroid–centroid separation is 3.7668 (9) Å and the inter-plane angle is 1.30 (7)°. This inter­action appears to be reinforced by a weak C—Cl⋯π_5_^ii^ bond (Chifotides & Dunbar, 2013 ▸); the chlorine atom lies almost directly above the centre of the six-membered ring displaced in [010] with Cl⋯π = 3.5363 (7) Å and C—Cl⋯π = 86.35 (5)°. This is very slightly shorter than the contact distance of 3.55 Å for a chlorine atom and a benzene ring, assuming a radius of 1.75 Å for Cl and a half-thickness of 1.8 Å for a benzene ring. Thus, each benzene ring faces a chlorine atom on one face and a five-membered ring on the other (Cl⋯π_6_⋯π_5_ = 154.5°). The carbonyl oxygen atom (O2) of the ester group lies in a reasonable orientation to partake in a C=O⋯π_5_ bond (Gao *et al.*, 2009 ▸) but here the O⋯π_5_^iii^ [symmetry code: (iii) *x*, *y* − 1, *z*] separation of 3.4068 (11) Å is significantly greater than the van der Waals’ radius sum of 3.32 Å [C=O⋯π_5_ = 88.40 (8)° and O⋯π_5_⋯π_6_ = 153.9°] and can hardly be considered to be a bond. Taken together, the strong (N—H⋯O) and weak (π–π, Cl⋯π) bonds lead to [010] double chains in the extended structure of (I).

Despite the fact that (I) and the Wu *et al.* (2013 ▸) polymorph of the same phase crystallize in the same space group, their packing motifs are completely different. In the Wu phase, inversion dimers linked by pairs of N—H⋯O hydrogen bonds also occur but there is no aromatic π–π stacking (the shortest centroid–centroid separation is greater than 4.75 Å) and no C—Cl⋯π contacts. The only significant inter­action indicated by a *PLATON* (Spek, 2009 ▸) analysis of the structure is a weak C—H⋯π_5_ bond (H⋯π = 2.72 Å). Considered by itself, this inter­action links the mol­ecules into [010] chains; taken together, the N—H⋯O and C—H⋯π inter­actions generate (110) sheets.

**Figure 4:**
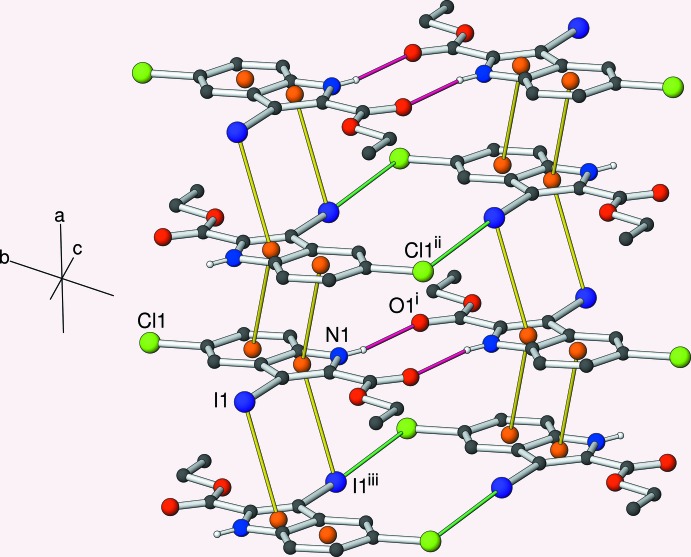
Partial packing diagram for (II), showing part of an (001) sheet. N—H⋯O hydrogen bonds are indicated by crimson lines, π–π and I⋯π inter­actions by yellow lines and I⋯Cl halogen bonds by green lines. All hydrogen atoms except H1 are omitted for clarity. Symmetry codes (i) −*x*, 2 − *y*, 1 − *z*; (ii) −*x*, 1 − *y*, 1 − *z*; (iii) 1 − *x*, 1 − *y*, 1 − *z*.

The crystal of (II) also features inversion dimers linked by pairs of N—H⋯O^i^ [symmetry code: (i) −*x*, 2 − *y*, 1 − *z*] hydrogen bonds (Table 2 ▸, Fig. 2 ▸) involving the equivalent atoms to (I) with the same graph-set motif. Aromatic π–π stacking also occurs in the crystal of (II), but this time the mol­ecules are related by inversion, rather than translation, symmetry: this operation ‘flips’ one of the mol­ecules such that the six-membered ring in each mol­ecule overlaps the five-membered ring in the other (Fig. 6): the π_6_–π_5_^ii^ [symmetry code: (ii) −*x*, 1 − *y*, 1 − *z*] separation of the centroids of the six- and five-membered rings is 3.6365 (14) Å and the inter-planar angle is 0.92 (13)°. The iodine atom of a mol­ecule displaced in the [100] direction lies above the inversion-generated five-membered ring to form a C—I⋯π_5_ bond with I1⋯π_5_^iii^ [symmetry code: (iii) 1 − *x*, 1 − *y*, 1 − *z*] = 3.6543 (11) Å and C7—I1⋯π_5_^iii^ = 87.00 (7)°. Thus, the five-membered ring faces a six-membered ring on one face and an I atom on the other (I⋯π_5_⋯π_6_ = 148.6°). The I atom also participates in a halogen bond (Desiraju *et al.*, 2013 ▸) to the chlorine atom of an inversion-related mol­ecule with I1⋯Cl1^iv^ [symmetry code: (iv) 1 − *x*, −*y*, 1 − *z*] = 3.6477 (6) Å (van der Waals contact distance = 3.73 Å), C7—I1⋯Cl1^iv^ = 173.28 (5)° and C4^iv^—Cl1^iv^⋯I1 = 104.34 (5)°. These angles clearly define this inter­action as a type-II halogen bond (Pedireddi *et al.*, 1994 ▸). Taken together, the weak and strong inter­actions lead to (001) sheets, with the centrosymmetric pairs of I⋯Cl halogen bonds and pairs of N—H⋯O hydrogen bonds alternating with respect to the [100] direction (Fig. 4 ▸).

## Database survey   

A search of the Cambridge Structural Database (CSD; Groom *et al.*, 2016 ▸) revealed 24 indole derivatives with an ester group at the 2-position of the ring system. In terms of halogen substitution, there were 58 5-chloro and just two 3-iodo deriv­atives. As noted above, VIHMUW (Wu *et al.*, 2013 ▸) is a polymorph of (I): crystals of this phase in the form of colourless prisms were obtained by recrystallization from ethanol solution at room temperature, compared to colourless needles obtained from methanol solution at room temperature in the present study.

There has recently been debate on the significance – or otherwise – of weak inter­molecular inter­actions in establishing the packing in mol­ecular crystals (Dunitz, 2015 ▸; Thakur *et al.*, 2015 ▸). The latter authors mentioned the role of weak inter­actions in establishing the structures of polymorphs and it is striking to us how different the packing motifs of (I) and VIHMUW are.

## Synthesis and crystallization   

To prepare (I), a mixture of ethyl 2-(2-[4-chloro­phen­yl]hydrazono)propano­ate (2.29 g, 9.51 mmol), prepared from *p*-chloro­phenyl­hydrazine hydro­chloride and ethyl pyruvate according to a published method (Zhang *et al.*, 2011 ▸) and PPA (22.54 g) were refluxed in toluene (40 ml) for 3 h. After cooling, the solvent was deca­nted off and the solid residue was washed with toluene (3 × 50 ml). Evaporation of the combined organic phases under reduced pressure gave a yellow solid, flash chromatography of which (1:6 ethyl acetate, hexa­nes) afforded ethyl 5-chloro-1*H*-indole-2-carboxyl­ate as a yellow solid (1.34 g, 63%). Colourless needles of (I) were recrystallized from methanol solution at room temperature. δC(101 MHz; CDCl_3_) 162.0 (Cq), 135.2 (Cq), 128.9 (Cq), 128.6 (Cq), 126.7 (Cq), 126.0 (CH), 121.9 (CH), 113.1 (CH), 108.1 (CH), 61.5 (CH_2_) and 14.5 (CH_3_); δH(400 MHz; CDCl_3_) 8.91 (1 H, *br s*), 7.67 (1 H, *s*), 7.35–7.28 (2 H, *m*), 7.15 (1 H, *s*), 4.41 (2 H, *q*, *J* 7.1) and 1.41 (3 H, *t*, *J* 7.1); *R*_f_ 0.29 (1:6 EtOAc, hexa­nes); m.p. 440–441 K; IR (Nujol, cm^−1^) 3310, 1728, 1697, 1264, 1080 and 877; HRMS (ESI) for C_11_H_11_^35^ClNO_2_ [*M* + H]^+^ calculated 224.0479, found 224.0466.

To prepare (II), potassium hydroxide (1.804 g, 32.2 mmol) was added to a solution of (I) (1.215 g, 5.43 mmol) in dry DMF (6.0 ml) at 273 K and stirred for 10 min. Separately, a solution of iodine (1.710 g, 6.74 mmol) in dry DMF (6.75 ml) was prepared. The two liquids were combined and stirred over ice for 90 min. Pouring the reaction mixture into a saturated aqueous solution of ammonium chloride and sodium thio­sulfate (60 ml) precipitated a brown solid. This was collected by filtration and purified by flash chromatography (1:8 ethyl acetate, hexa­nes) to afford ethyl 5-chloro-3-iodo-1*H*-indole-2-carboxyl­ate as a yellow solid (1.825 g, 80%). Pale-yellow plates of (II) were recrystallized from methanol solution at room temperature. δC(101 MHz; DMSO-*d*_6_) 160.5 (Cq), 135.8 (Cq), 132.1 (Cq), 128.9 (Cq), 126.5 (CH), 126.3 (Cq), 121.8 (CH), 115.4 (CH), 65.2 (Cq), 61.4 (CH_2_) and 14.6 (CH_3_); δH(400 MHz; DMSO-*d*_6_) 12.42 (1 H, *br s*), 7.47 (1 H, *d*, *J* 8.4), 7.39 (1 H, *d*, *J* 1.6), 7.31 (1 H, *dd*, *J* 2.0, 9.2), 4.36 (2 H, *q*, *J* 7.2) and 1.36 (3 H, *t*, *J* 7.0); *R*_f_ 0.13 (1:8 ethyl acetate, hexa­nes); m.p. 412 K, IR (KBr, cm^−1^) 3291, 2977, 1744, 1683, 1514, 1332, 1115, 1080, 772, 749 and 604; HRMS (ESI) for C_11_H_10_^35^ClINO_2_ [*M* + H]^+^ calculated 349.9445, found 349.9453.

## Refinement   

Crystal data, data collection and structure refinement details are summarized in Table 3 ▸. The N-bound H atoms were located in difference maps and their positions freely refined. The C-bound H atoms were placed geometrically (C—H = 0.93–0.98 Å) and refined as riding atoms. The constraint *U*_iso_(H) = 1.2*U*_eq_(carrier) or 1.5*U*_eq_(methyl carrier) was applied in all cases. The –CH_3_ groups were allowed to rotate, but not to tip, to best fit the electron density.

## Supplementary Material

Crystal structure: contains datablock(s) I, II, global. DOI: 10.1107/S2056989016008616/sj5499sup1.cif

Structure factors: contains datablock(s) I. DOI: 10.1107/S2056989016008616/sj5499Isup2.hkl

Structure factors: contains datablock(s) II. DOI: 10.1107/S2056989016008616/sj5499IIsup3.hkl

Supporting information file. DOI: 10.1107/S2056989016008616/sj5499Isup4.cml

Supporting information file. DOI: 10.1107/S2056989016008616/sj5499IIsup5.cml

CCDC references: 1482360, 1482359

Additional supporting information:  crystallographic information; 3D view; checkCIF report

## Figures and Tables

**Table 1:** Hydrogen-bond geometry (Å, °) for (I)

*D*—H⋯*A*	*D*—H	H⋯*A*	*D*⋯*A*	*D*—H⋯*A*
N1—H1⋯O2^i^	0.878 (17)	1.977 (17)	2.8288 (15)	163.0 (15)

Symmetry code: (i) 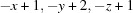.

**Table 2:** Hydrogen-bond geometry (Å, °) for (II)

*D*—H⋯*A*	*D*—H	H⋯*A*	*D*⋯*A*	*D*—H⋯*A*
N1—H1⋯O2^i^	0.77 (3)	2.06 (3)	2.821 (2)	168 (3)

Symmetry code: (i) 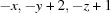.

**Table 3:** Experimental details

	(I)	(II)
Crystal data		
Chemical formula	C_11_H_10_ClNO_2_	C_11_H_9_ClINO_2_
*M*_r_	223.65	349.54
Crystal system, space group	Monoclinic, *P*2_1_/*n*	Triclinic, *P* 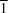
Temperature (K)	100	100
*a*, *b*, *c* (Å)	13.7168 (6), 4.5783 (1), 16.5929 (11)	7.7733 (5), 7.8240 (5), 10.4594 (7)
α, β, γ (°)	90, 97.464 (7), 90	86.085 (8), 80.575 (7), 71.308 (6)
*V* (Å^3^)	1033.20 (9)	594.35 (7)
*Z*	4	2
Radiation type	Mo *K*α	Mo *K*α
μ (mm^−1^)	0.35	2.90
Crystal size (mm)	0.70 × 0.04 × 0.03	0.17 × 0.10 × 0.02

Data collection		
Diffractometer	Rigaku Mercury CCD	Rigaku Mercury CCD
Absorption correction	Multi-scan (*CrystalClear*; Rigaku, 2012 ▸)	Multi-scan (*CrystalClear*; Rigaku, 2012 ▸)
*T*_min_, *T*_max_	0.793, 0.990	0.638, 0.944
No. of measured, independent and observed [*I* > 2σ(*I*)] reflections	7035, 2340, 2051	7837, 2739, 2640
*R*_int_	0.022	0.031
(sin θ/λ)_max_ (Å^−1^)	0.649	0.650

Refinement		
*R*[*F* ^2^ > 2σ(*F* ^2^)], *wR*(*F* ^2^), *S*	0.031, 0.082, 1.09	0.022, 0.058, 1.04
No. of reflections	2340	2739
No. of parameters	139	149
H-atom treatment	H atoms treated by a mixture of independent and constrained refinement	H atoms treated by a mixture of independent and constrained refinement
Δρ_max_, Δρ_min_ (e Å^−3^)	0.33, −0.23	1.18, −0.44

Computer programs: *CrystalClear* (Rigaku, 2012 ▸), *SHELXS97* (Sheldrick, 2008 ▸), *SHELXL2014* (Sheldrick, 2015 ▸), *ORTEP-3 for Windows* (Farrugia, 2012 ▸), *ATOMS* (Dowty, 2006 ▸) and *publCIF* (Westrip, 2010 ▸).

## supplementary crystallographic information

### (I) Ethyl 5-chloro-1H-indole-2-carboxylate Crystal data

**Table:**

C_11_H_10_ClNO_2_	*F*(000) = 464
*M**_r_* = 223.65	*D*_x_ = 1.438 Mg m^−^^3^
Monoclinic, *P*2_1_/*n*	Mo *K*α radiation, λ = 0.71073 Å
*a* = 13.7168 (6) Å	Cell parameters from 6396 reflections
*b* = 4.5783 (1) Å	θ = 3.0–27.5°
*c* = 16.5929 (11) Å	µ = 0.35 mm^−^^1^
β = 97.464 (7)°	*T* = 100 K
*V* = 1033.20 (9) Å^3^	Rod, colourless
*Z* = 4	0.70 × 0.04 × 0.03 mm

### (I) Ethyl 5-chloro-1H-indole-2-carboxylate Data collection

**Table:**

Rigaku Mercury CCD diffractometer	2051 reflections with *I* > 2σ(*I*)
ω scans	*R*_int_ = 0.022
Absorption correction: multi-scan (CrystalClear; Rigaku, 2012)	θ_max_ = 27.5°, θ_min_ = 3.0°
*T*_min_ = 0.793, *T*_max_ = 0.990	*h* = −17→17
7035 measured reflections	*k* = −4→5
2340 independent reflections	*l* = −19→21

### (I) Ethyl 5-chloro-1H-indole-2-carboxylate Refinement

**Table:**

Refinement on *F*^2^	Primary atom site location: structure-invariant direct methods
Least-squares matrix: full	Hydrogen site location: mixed
*R*[*F*^2^ > 2σ(*F*^2^)] = 0.031	H atoms treated by a mixture of independent and constrained refinement
*wR*(*F*^2^) = 0.082	*w* = 1/[σ^2^(*F*_o_^2^) + (0.0408*P*)^2^ + 0.4177*P*] where *P* = (*F*_o_^2^ + 2*F*_c_^2^)/3
*S* = 1.09	(Δ/σ)_max_ < 0.001
2340 reflections	Δρ_max_ = 0.33 e Å^−^^3^
139 parameters	Δρ_min_ = −0.23 e Å^−^^3^
0 restraints	

### (I) Ethyl 5-chloro-1H-indole-2-carboxylate Special details

**Table:**

Geometry. All esds (except the esd in the dihedral angle between two l.s. planes) are estimated using the full covariance matrix. The cell esds are taken into account individually in the estimation of esds in distances, angles and torsion angles; correlations between esds in cell parameters are only used when they are defined by crystal symmetry. An approximate (isotropic) treatment of cell esds is used for estimating esds involving l.s. planes.

### (I) Ethyl 5-chloro-1H-indole-2-carboxylate Fractional atomic coordinates and isotropic or equivalent isotropic displacement parameters (Å^2^)

**Table:**

	*x*	*y*	*z*	*U*_iso_*/*U*_eq_	
C1	0.52594 (9)	0.5040 (3)	0.35005 (8)	0.0142 (3)	
C2	0.62255 (10)	0.4033 (3)	0.35004 (8)	0.0176 (3)	
H2	0.6742	0.4669	0.3899	0.021*	
C3	0.63988 (9)	0.2090 (3)	0.29013 (8)	0.0171 (3)	
H3	0.7044	0.1360	0.2883	0.021*	
C4	0.56188 (10)	0.1181 (3)	0.23127 (8)	0.0153 (3)	
C5	0.46670 (9)	0.2127 (3)	0.23045 (8)	0.0146 (3)	
H5	0.4157	0.1459	0.1905	0.017*	
C6	0.44761 (9)	0.4121 (3)	0.29091 (8)	0.0134 (3)	
C7	0.36151 (9)	0.5605 (3)	0.30827 (8)	0.0136 (3)	
H7	0.2972	0.5434	0.2795	0.016*	
C8	0.38999 (9)	0.7341 (3)	0.37519 (7)	0.0135 (3)	
C9	0.33337 (9)	0.9388 (3)	0.41806 (7)	0.0133 (3)	
C10	0.17639 (10)	1.1516 (3)	0.42258 (8)	0.0174 (3)	
H10A	0.1186	1.2025	0.3829	0.021*	
H10B	0.2134	1.3334	0.4376	0.021*	
C11	0.14202 (11)	1.0204 (4)	0.49746 (9)	0.0271 (3)	
H11A	0.1002	1.1610	0.5214	0.041*	
H11B	0.1044	0.8421	0.4825	0.041*	
H11C	0.1991	0.9728	0.5371	0.041*	
N1	0.48871 (8)	0.6981 (3)	0.40047 (7)	0.0147 (2)	
H1	0.5235 (12)	0.789 (4)	0.4411 (10)	0.018*	
O1	0.23886 (6)	0.9493 (2)	0.38517 (5)	0.0152 (2)	
O2	0.36753 (7)	1.0841 (2)	0.47654 (6)	0.0184 (2)	
Cl1	0.58935 (2)	−0.12275 (8)	0.15557 (2)	0.01929 (11)	

### (I) Ethyl 5-chloro-1H-indole-2-carboxylate Atomic displacement parameters (Å^2^)

**Table:**

	*U*^11^	*U*^22^	*U*^33^	*U*^12^	*U*^13^	*U*^23^
C1	0.0137 (6)	0.0138 (6)	0.0147 (6)	−0.0016 (5)	−0.0002 (5)	0.0003 (5)
C2	0.0128 (6)	0.0196 (7)	0.0193 (6)	−0.0010 (5)	−0.0026 (5)	−0.0010 (5)
C3	0.0123 (6)	0.0175 (7)	0.0213 (7)	0.0013 (5)	0.0012 (5)	0.0009 (6)
C4	0.0175 (6)	0.0126 (6)	0.0164 (6)	−0.0001 (5)	0.0040 (5)	−0.0007 (5)
C5	0.0144 (6)	0.0146 (6)	0.0140 (6)	−0.0017 (5)	−0.0008 (5)	0.0004 (5)
C6	0.0134 (6)	0.0126 (6)	0.0135 (6)	−0.0014 (5)	−0.0007 (5)	0.0020 (5)
C7	0.0130 (6)	0.0140 (6)	0.0134 (6)	−0.0011 (5)	−0.0004 (4)	0.0008 (5)
C8	0.0122 (6)	0.0149 (6)	0.0129 (6)	−0.0012 (5)	−0.0007 (4)	0.0014 (5)
C9	0.0142 (6)	0.0132 (6)	0.0122 (6)	−0.0014 (5)	0.0007 (4)	0.0025 (5)
C10	0.0149 (6)	0.0169 (7)	0.0203 (7)	0.0043 (5)	0.0013 (5)	−0.0010 (5)
C11	0.0248 (7)	0.0318 (9)	0.0265 (8)	0.0035 (7)	0.0106 (6)	0.0017 (7)
N1	0.0125 (5)	0.0175 (6)	0.0130 (5)	−0.0004 (5)	−0.0023 (4)	−0.0027 (4)
O1	0.0126 (4)	0.0171 (5)	0.0154 (4)	0.0015 (4)	−0.0005 (3)	−0.0023 (4)
O2	0.0164 (5)	0.0218 (5)	0.0158 (5)	−0.0001 (4)	−0.0018 (4)	−0.0050 (4)
Cl1	0.01786 (17)	0.01983 (19)	0.02054 (18)	0.00168 (13)	0.00388 (12)	−0.00493 (13)

### (I) Ethyl 5-chloro-1H-indole-2-carboxylate Geometric parameters (Å, º)

**Table:**

C1—N1	1.3644 (18)	C7—H7	0.9500
C1—C2	1.4032 (18)	C8—N1	1.3744 (16)
C1—C6	1.4215 (17)	C8—C9	1.4599 (19)
C2—C3	1.3774 (19)	C9—O2	1.2187 (16)
C2—H2	0.9500	C9—O1	1.3405 (15)
C3—C4	1.4145 (19)	C10—O1	1.4543 (16)
C3—H3	0.9500	C10—C11	1.5098 (19)
C4—C5	1.3739 (18)	C10—H10A	0.9900
C4—Cl1	1.7488 (14)	C10—H10B	0.9900
C5—C6	1.4059 (18)	C11—H11A	0.9800
C5—H5	0.9500	C11—H11B	0.9800
C6—C7	1.4240 (18)	C11—H11C	0.9800
C7—C8	1.3800 (18)	N1—H1	0.878 (17)
			
N1—C1—C2	130.00 (12)	N1—C8—C9	119.57 (11)
N1—C1—C6	107.86 (11)	C7—C8—C9	130.46 (12)
C2—C1—C6	122.13 (13)	O2—C9—O1	123.78 (12)
C3—C2—C1	117.63 (12)	O2—C9—C8	124.37 (12)
C3—C2—H2	121.2	O1—C9—C8	111.85 (11)
C1—C2—H2	121.2	O1—C10—C11	111.24 (12)
C2—C3—C4	120.17 (12)	O1—C10—H10A	109.4
C2—C3—H3	119.9	C11—C10—H10A	109.4
C4—C3—H3	119.9	O1—C10—H10B	109.4
C5—C4—C3	123.13 (12)	C11—C10—H10B	109.4
C5—C4—Cl1	119.00 (10)	H10A—C10—H10B	108.0
C3—C4—Cl1	117.87 (10)	C10—C11—H11A	109.5
C4—C5—C6	117.55 (12)	C10—C11—H11B	109.5
C4—C5—H5	121.2	H11A—C11—H11B	109.5
C6—C5—H5	121.2	C10—C11—H11C	109.5
C5—C6—C1	119.38 (12)	H11A—C11—H11C	109.5
C5—C6—C7	133.66 (12)	H11B—C11—H11C	109.5
C1—C6—C7	106.95 (11)	C1—N1—C8	108.85 (11)
C8—C7—C6	106.38 (11)	C1—N1—H1	124.7 (11)
C8—C7—H7	126.8	C8—N1—H1	126.4 (11)
C6—C7—H7	126.8	C9—O1—C10	116.19 (10)
N1—C8—C7	109.96 (12)		
			
N1—C1—C2—C3	−178.66 (14)	C1—C6—C7—C8	0.37 (15)
C6—C1—C2—C3	−0.1 (2)	C6—C7—C8—N1	−0.63 (15)
C1—C2—C3—C4	0.2 (2)	C6—C7—C8—C9	178.10 (13)
C2—C3—C4—C5	−0.5 (2)	N1—C8—C9—O2	−0.4 (2)
C2—C3—C4—Cl1	178.63 (11)	C7—C8—C9—O2	−178.99 (14)
C3—C4—C5—C6	0.8 (2)	N1—C8—C9—O1	179.38 (11)
Cl1—C4—C5—C6	−178.38 (10)	C7—C8—C9—O1	0.8 (2)
C4—C5—C6—C1	−0.67 (19)	C2—C1—N1—C8	178.35 (14)
C4—C5—C6—C7	178.26 (14)	C6—C1—N1—C8	−0.40 (15)
N1—C1—C6—C5	179.20 (11)	C7—C8—N1—C1	0.65 (15)
C2—C1—C6—C5	0.3 (2)	C9—C8—N1—C1	−178.23 (12)
N1—C1—C6—C7	0.01 (15)	O2—C9—O1—C10	0.89 (18)
C2—C1—C6—C7	−178.85 (12)	C8—C9—O1—C10	−178.86 (11)
C5—C6—C7—C8	−178.65 (14)	C11—C10—O1—C9	−81.73 (14)

### (I) Ethyl 5-chloro-1H-indole-2-carboxylate Hydrogen-bond geometry (Å, º)

**Table:**

*D*—H···*A*	*D*—H	H···*A*	*D*···*A*	*D*—H···*A*
N1—H1···O2^i^	0.878 (17)	1.977 (17)	2.8288 (15)	163.0 (15)

Symmetry code: (i) −*x*+1, −*y*+2, −*z*+1.

### (II) Ethyl 5-chloro-3-iodo-1H-indole-2-carboxylate Crystal data

**Table:**

C_11_H_9_ClINO_2_	*Z* = 2
*M**_r_* = 349.54	*F*(000) = 336
Triclinic, *P*1	*D*_x_ = 1.953 Mg m^−^^3^
*a* = 7.7733 (5) Å	Mo *K*α radiation, λ = 0.71073 Å
*b* = 7.8240 (5) Å	Cell parameters from 7985 reflections
*c* = 10.4594 (7) Å	θ = 2.7–27.5°
α = 86.085 (8)°	µ = 2.90 mm^−^^1^
β = 80.575 (7)°	*T* = 100 K
γ = 71.308 (6)°	Plate, colourless
*V* = 594.35 (7) Å^3^	0.17 × 0.10 × 0.02 mm

### (II) Ethyl 5-chloro-3-iodo-1H-indole-2-carboxylate Data collection

**Table:**

Rigaku Mercury CCD diffractometer	2640 reflections with *I* > 2σ(*I*)
ω scans	*R*_int_ = 0.031
Absorption correction: multi-scan (CrystalClear; Rigaku, 2012)	θ_max_ = 27.5°, θ_min_ = 2.8°
*T*_min_ = 0.638, *T*_max_ = 0.944	*h* = −8→10
7837 measured reflections	*k* = −10→10
2739 independent reflections	*l* = −12→13

### (II) Ethyl 5-chloro-3-iodo-1H-indole-2-carboxylate Refinement

**Table:**

Refinement on *F*^2^	0 restraints
Least-squares matrix: full	Hydrogen site location: mixed
*R*[*F*^2^ > 2σ(*F*^2^)] = 0.022	H atoms treated by a mixture of independent and constrained refinement
*wR*(*F*^2^) = 0.058	*w* = 1/[σ^2^(*F*_o_^2^) + (0.0409*P*)^2^ + 0.1325*P*] where *P* = (*F*_o_^2^ + 2*F*_c_^2^)/3
*S* = 1.04	(Δ/σ)_max_ = 0.002
2739 reflections	Δρ_max_ = 1.18 e Å^−^^3^
149 parameters	Δρ_min_ = −0.44 e Å^−^^3^

### (II) Ethyl 5-chloro-3-iodo-1H-indole-2-carboxylate Special details

**Table:**

Geometry. All esds (except the esd in the dihedral angle between two l.s. planes) are estimated using the full covariance matrix. The cell esds are taken into account individually in the estimation of esds in distances, angles and torsion angles; correlations between esds in cell parameters are only used when they are defined by crystal symmetry. An approximate (isotropic) treatment of cell esds is used for estimating esds involving l.s. planes.

### (II) Ethyl 5-chloro-3-iodo-1H-indole-2-carboxylate Fractional atomic coordinates and isotropic or equivalent isotropic displacement parameters (Å^2^)

**Table:**

	*x*	*y*	*z*	*U*_iso_*/*U*_eq_	
C1	0.0847 (3)	0.5746 (3)	0.6457 (2)	0.0165 (4)	
C2	−0.0166 (3)	0.5738 (3)	0.7698 (2)	0.0190 (4)	
H2	−0.0956	0.6824	0.8097	0.023*	
C3	0.0037 (3)	0.4078 (3)	0.8316 (2)	0.0204 (4)	
H3	−0.0631	0.4013	0.9154	0.024*	
C4	0.1227 (3)	0.2486 (3)	0.7712 (2)	0.0185 (4)	
C5	0.2249 (3)	0.2468 (3)	0.6505 (2)	0.0176 (4)	
H5	0.3045	0.1374	0.6122	0.021*	
C6	0.2064 (3)	0.4142 (3)	0.5863 (2)	0.0160 (4)	
C7	0.2860 (3)	0.4665 (3)	0.46397 (19)	0.0144 (4)	
C8	0.2117 (3)	0.6518 (3)	0.45207 (19)	0.0155 (4)	
C9	0.2427 (3)	0.7849 (3)	0.3534 (2)	0.0162 (4)	
C10	0.4005 (3)	0.8407 (3)	0.1517 (2)	0.0196 (4)	
H10A	0.4584	0.9185	0.1879	0.023*	
H10B	0.2863	0.9183	0.1211	0.023*	
C11	0.5308 (3)	0.7311 (3)	0.0411 (2)	0.0260 (5)	
H11A	0.5687	0.8125	−0.0247	0.039*	
H11B	0.4686	0.6612	0.0024	0.039*	
H11C	0.6392	0.6488	0.0740	0.039*	
N1	0.0899 (2)	0.7153 (2)	0.56278 (17)	0.0159 (3)	
H1	0.030 (4)	0.814 (4)	0.576 (3)	0.019*	
O1	0.3601 (2)	0.71212 (19)	0.24936 (14)	0.0179 (3)	
O2	0.1685 (2)	0.9465 (2)	0.36622 (16)	0.0232 (3)	
Cl1	0.14126 (8)	0.04416 (8)	0.85625 (5)	0.02534 (12)	
I1	0.48122 (2)	0.28666 (2)	0.33716 (2)	0.01543 (6)	

### (II) Ethyl 5-chloro-3-iodo-1H-indole-2-carboxylate Atomic displacement parameters (Å^2^)

**Table:**

	*U*^11^	*U*^22^	*U*^33^	*U*^12^	*U*^13^	*U*^23^
C1	0.0128 (9)	0.0169 (9)	0.0192 (10)	−0.0027 (7)	−0.0041 (7)	−0.0020 (8)
C2	0.0143 (9)	0.0213 (10)	0.0191 (10)	−0.0020 (8)	−0.0017 (7)	−0.0036 (8)
C3	0.0156 (9)	0.0278 (11)	0.0163 (10)	−0.0053 (8)	−0.0015 (7)	0.0002 (8)
C4	0.0171 (9)	0.0168 (9)	0.0207 (10)	−0.0044 (8)	−0.0049 (8)	0.0049 (8)
C5	0.0139 (9)	0.0160 (9)	0.0207 (10)	−0.0013 (8)	−0.0038 (7)	0.0001 (8)
C6	0.0135 (9)	0.0170 (9)	0.0168 (9)	−0.0027 (7)	−0.0038 (7)	−0.0015 (7)
C7	0.0126 (8)	0.0125 (8)	0.0171 (9)	−0.0024 (7)	−0.0021 (7)	−0.0009 (7)
C8	0.0129 (8)	0.0163 (9)	0.0169 (9)	−0.0031 (7)	−0.0033 (7)	−0.0016 (7)
C9	0.0144 (9)	0.0143 (9)	0.0195 (10)	−0.0038 (7)	−0.0025 (7)	−0.0004 (7)
C10	0.0222 (10)	0.0162 (9)	0.0197 (10)	−0.0068 (8)	−0.0013 (8)	0.0043 (8)
C11	0.0347 (12)	0.0227 (11)	0.0205 (11)	−0.0121 (10)	0.0024 (9)	−0.0009 (8)
N1	0.0139 (8)	0.0129 (8)	0.0186 (8)	−0.0010 (6)	−0.0016 (6)	−0.0018 (6)
O1	0.0206 (7)	0.0125 (6)	0.0188 (7)	−0.0044 (6)	−0.0001 (6)	0.0020 (5)
O2	0.0240 (8)	0.0135 (7)	0.0268 (8)	−0.0018 (6)	0.0023 (6)	−0.0001 (6)
Cl1	0.0254 (3)	0.0228 (3)	0.0242 (3)	−0.0060 (2)	−0.0008 (2)	0.0092 (2)
I1	0.01470 (9)	0.01248 (9)	0.01706 (9)	−0.00172 (6)	−0.00130 (6)	−0.00153 (6)

### (II) Ethyl 5-chloro-3-iodo-1H-indole-2-carboxylate Geometric parameters (Å, º)

**Table:**

C1—N1	1.361 (3)	C7—I1	2.0660 (19)
C1—C2	1.405 (3)	C8—N1	1.380 (3)
C1—C6	1.417 (3)	C8—C9	1.463 (3)
C2—C3	1.384 (3)	C9—O2	1.216 (3)
C2—H2	0.9500	C9—O1	1.330 (2)
C3—C4	1.409 (3)	C10—O1	1.453 (2)
C3—H3	0.9500	C10—C11	1.515 (3)
C4—C5	1.376 (3)	C10—H10A	0.9900
C4—Cl1	1.752 (2)	C10—H10B	0.9900
C5—C6	1.406 (3)	C11—H11A	0.9800
C5—H5	0.9500	C11—H11B	0.9800
C6—C7	1.421 (3)	C11—H11C	0.9800
C7—C8	1.383 (3)	N1—H1	0.77 (3)
			
N1—C1—C2	129.80 (19)	N1—C8—C9	117.43 (17)
N1—C1—C6	108.08 (18)	C7—C8—C9	133.80 (19)
C2—C1—C6	122.11 (19)	O2—C9—O1	123.5 (2)
C3—C2—C1	117.05 (19)	O2—C9—C8	122.96 (19)
C3—C2—H2	121.5	O1—C9—C8	113.50 (17)
C1—C2—H2	121.5	O1—C10—C11	106.61 (17)
C2—C3—C4	120.58 (19)	O1—C10—H10A	110.4
C2—C3—H3	119.7	C11—C10—H10A	110.4
C4—C3—H3	119.7	O1—C10—H10B	110.4
C5—C4—C3	123.23 (19)	C11—C10—H10B	110.4
C5—C4—Cl1	119.08 (16)	H10A—C10—H10B	108.6
C3—C4—Cl1	117.69 (16)	C10—C11—H11A	109.5
C4—C5—C6	117.06 (19)	C10—C11—H11B	109.5
C4—C5—H5	121.5	H11A—C11—H11B	109.5
C6—C5—H5	121.5	C10—C11—H11C	109.5
C5—C6—C1	119.95 (19)	H11A—C11—H11C	109.5
C5—C6—C7	133.51 (19)	H11B—C11—H11C	109.5
C1—C6—C7	106.53 (18)	C1—N1—C8	109.37 (17)
C8—C7—C6	107.32 (17)	C1—N1—H1	124 (2)
C8—C7—I1	129.30 (15)	C8—N1—H1	127 (2)
C6—C7—I1	123.36 (14)	C9—O1—C10	115.06 (16)
N1—C8—C7	108.70 (18)		
			
N1—C1—C2—C3	179.0 (2)	C1—C6—C7—I1	178.14 (13)
C6—C1—C2—C3	−1.6 (3)	C6—C7—C8—N1	0.2 (2)
C1—C2—C3—C4	0.5 (3)	I1—C7—C8—N1	−178.26 (13)
C2—C3—C4—C5	0.4 (3)	C6—C7—C8—C9	176.9 (2)
C2—C3—C4—Cl1	179.75 (16)	I1—C7—C8—C9	−1.5 (3)
C3—C4—C5—C6	−0.2 (3)	N1—C8—C9—O2	1.2 (3)
Cl1—C4—C5—C6	−179.59 (15)	C7—C8—C9—O2	−175.3 (2)
C4—C5—C6—C1	−0.8 (3)	N1—C8—C9—O1	−179.20 (16)
C4—C5—C6—C7	−179.7 (2)	C7—C8—C9—O1	4.3 (3)
N1—C1—C6—C5	−178.72 (18)	C2—C1—N1—C8	179.1 (2)
C2—C1—C6—C5	1.7 (3)	C6—C1—N1—C8	−0.4 (2)
N1—C1—C6—C7	0.5 (2)	C7—C8—N1—C1	0.1 (2)
C2—C1—C6—C7	−179.05 (18)	C9—C8—N1—C1	−177.20 (17)
C5—C6—C7—C8	178.7 (2)	O2—C9—O1—C10	2.2 (3)
C1—C6—C7—C8	−0.4 (2)	C8—C9—O1—C10	−177.42 (16)
C5—C6—C7—I1	−2.8 (3)	C11—C10—O1—C9	−178.33 (17)

### (II) Ethyl 5-chloro-3-iodo-1H-indole-2-carboxylate Hydrogen-bond geometry (Å, º)

**Table:**

*D*—H···*A*	*D*—H	H···*A*	*D*···*A*	*D*—H···*A*
N1—H1···O2^i^	0.77 (3)	2.06 (3)	2.821 (2)	168 (3)

Symmetry code: (i) −*x*, −*y*+2, −*z*+1.

## References

[bb1] Chifotides, H. T. & Dunbar, K. R. (2013). *Acc. Chem. Res.* **46**, 894–906. 10.1021/ar300251k 23477406

[bb2] Desiraju, G. R., Ho, P. S., Kloo, L., Legon, A. C., Marquardt, R., Metrangolo, P., Politzer, P., Resnati, G. & Rissanen, K. (2013). *Pure Appl. Chem.* **85**, 1711–1713.

[bb3] Dowty, E. W. (2006). *ATOMS*. Shape Software, Kingsport, Tennessee, USA.

[bb4] Dunitz, J. D. (2015). *IUCrJ*, **2**, 157–158. 10.1107/S2052252515002006 PMC4392407 25866649

[bb5] Farrugia, L. J. (2012). *J. Appl. Cryst.* **45**, 849–854.

[bb6] Gao, X.-L., Lu, L.-P. & Zhu, M.-L. (2009). *Acta Cryst.* C**65**, o123–o127. 10.1107/S0108270109005836 19346605

[bb7] Groom, C. R., Bruno, I. J., Lightfoot, M. P. & Ward, S. C. (2016). *Acta Cryst.* B**72**, 171–179. 10.1107/S2052520616003954 PMC4822653 27048719

[bb8] Kerr, J. R. (2013). PhD Thesis ‘Allosteric modulation of the CB1 receptor’. University of Aberdeen.

[bb9] Kerr, J. R., Trembleau, L., Storey, J. M. D., Wardell, J. L. & Harrison, W. T. A. (2016). *Acta Cryst.* E**72**, 699–703. 10.1107/S2056989016006162 PMC4908542 27308022

[bb10] Pedireddi, V. R., Reddy, D. S., Goud, B. S., Craig, D. C., Rae, A. D. & Desiraju, G. R. (1994). *J. Chem. Soc. Perkin Trans. 2*, pp. 2353–2360.

[bb11] Rigaku (2012). *CrystalClear*. Rigaku Corporation, Tokyo, Japan.

[bb12] Sheldrick, G. M. (2008). *Acta Cryst.* A**64**, 112–122. 10.1107/S0108767307043930 18156677

[bb13] Sheldrick, G. M. (2015). *Acta Cryst.* C**71**, 3–8.

[bb14] Spek, A. L. (2009). *Acta Cryst* D**65**, 148–155. 10.1107/S090744490804362X PMC2631630 19171970

[bb15] Thakur, T. S., Dubey, R. & Desiraju, G. R. (2015). *IUCrJ*, **2**, 159–160. 10.1107/S205225251500189X PMC4392408 25866650

[bb16] Westrip, S. P. (2010). *J. Appl. Cryst.* **43**, 920–925.

[bb17] Wu, J., Liu, Y.-L., Wu, H., Wang, H.-Y. & Zou, P. (2013). *Z. Krist. New Cryst. Struct.* **228**, 185–186.

[bb18] Zhang, F., Zhao, Y., Sun, L., Ding, L., Gu, Y. & Gong, P. (2011). *Eur. J. Med. Chem.* **46**, 3149–3157. 10.1016/j.ejmech.2011.03.055 21514012

